# RBP-J is required for M2 macrophage polarization in response to chitin and mediates expression of a subset of M2 genes

**DOI:** 10.1007/s13238-016-0248-7

**Published:** 2016-02-13

**Authors:** Julia Foldi, Yingli Shang, Baohong Zhao, Lionel B. Ivashkiv, Xiaoyu Hu

**Affiliations:** Graduate Program in Immunology and Microbial Pathogenesis, Weill Cornell Graduate School of Medical Sciences, New York, NY 10021 USA; School of Medicine and Institute for Immunology, Tsinghua University, Beijing, 100084 China; Arthritis and Tissue Degeneration Program, Hospital for Special Surgery, New York, NY 10021 USA

**Keywords:** macrophages, RBP-J, M2, arginase, chitin

## Abstract

Development of alternatively activated (M2) macrophage phenotypes is a complex process that is coordinately regulated by a plethora of pathways and factors. Here, we report that RBP-J, a DNA-binding protein that integrates signals from multiple pathways including the Notch pathway, is critically involved in polarization of M2 macrophages. Mice deficient in RBP-J in the myeloid compartment exhibited impaired M2 phenotypes *in vivo* in a chitin-induced model of M2 polarization. Consistent with the *in vivo* findings, M2 polarization was partially compromised *in vitro* in *Rbpj*-deficient macrophages as demonstrated by reduced expression of a subset of M2 effector molecules including arginase 1. Functionally, myeloid *Rbpj* deficiency impaired M2 effector functions including recruitment of eosinophils and suppression of T cell proliferation. Collectively, we have identified RBP-J as an essential regulator of differentiation and function of alternatively activated macrophages.

## Introduction

Macrophages are versatile cells with diverse functions in inflammation, tissue remodeling, angiogenesis, and tumor immunity. They respond to a wide variety of environmental cues to regulate immunity and inflammation by sensing microbial pathogens, secreting cytokines and inflammatory mediators, and presenting antigens to T cells. Depending on environmental signals, macrophages can display a spectrum of activation states ranging from classically activated, M1 inflammatory macrophages, to various alternatively activated M2 macrophages that are involved in immune regulation and tissue repair (Murray and Wynn, 2011; Ivashkiv, 2013). M1 macrophages are characterized by production of high levels of inflammatory mediators in response to stimulation by pathogen-associated molecular patterns and/or inflammatory cytokines. In contrast, M2 macrophages express less inflammatory mediators and play a key role in wound healing and resolution of inflammation (Mosser and Edwards, 2008; Murray et al., 2014). One mechanism by which M2 macrophages limit inflammation and restore homeostasis is suppression of T cell proliferation, which is mediated at least in part by a key M2 effector molecule arginase 1 (Arg1) (Chawla et al., 2011). Besides their suppressive functions, M2 macrophages are also involved in host defense against helminthes by recruiting eosinophils to the sites of infection. M2 polarization is driven by products of mast cells, T_H_2 cells, and basophils including IL-4 that signals through the Jak-STAT signaling pathway to activate the latent transcription factor STAT6 (Chawla et al., 2011). IL-4-signaling promotes M2 polarization by upregulating expression of genes including those encoding Arg1, mannose receptor (MR), and Ym1, hallmarks of M2 phenotypes. In addition to IL-4-STAT6, a number of pathways and transcription factors such as PPARγ and IRF4 are also critically involved in driving M2 polarization (Odegaard et al., 2007; Satoh et al., 2010).

Recombinant recognition sequence binding protein at the Jκ site (RBP-J, also named CSL or CBF1) is the central nuclear mediator of canonical Notch signaling whose activation leads to transcription of Notch target genes. The evolutionarily conserved Notch signaling pathway regulates cell proliferation, apoptosis, and cell fate decisions during development and adult tissue homeostasis (Kopan and Ilagan, 2009). Recent studies using global expression analysis and chromatin immunoprecipitation deep-sequencing (ChIP-seq) have revealed genome-wide Notch-RBP-J targets in various systems including hematopoiesis (Hamidi et al., 2011), neural stem cell differentiation (Li et al., 2012), Epstein-Barr virus infection (Zhao et al., 2011), and T-lymhoblastic leukemia/lymphoma (Palomero et al., 2006; Wang et al., 2011). In the immune system, the most established functions for Notch signaling is regulation of development of lymphoid T and B cell lineages (Radtke et al., 2010; Yuan et al., 2010). Less well characterized, however, is the role of Notch signaling in myeloid cell differentiation and function. Notch-RBP-J signaling has been shown to control development and differentiation of multiple myeloid lineages including granulocyte/monocyte progenitors, dendritic cells, and osteoclasts (Klinakis et al., 2011; Lewis et al., 2011; Zhao et al., 2012). Although Notch signaling is not required for normal development of macrophage populations under homeostatic conditions, recently mounting evidence suggests that Notch signaling has profound effects on macrophage activation and polarization. Constitutive expression of Notch pathway components and constitutive activities of Notch signaling have been detected on macrophages of both human and mouse origin (Monsalve et al., 2006; Fung et al., 2007; Hu et al., 2008; Zhang et al., 2010). Notch signaling in macrophage regulates expression of pro- and anti-inflammatory mediators in response to macrophage activating signals such as LPS (Hu et al., 2008; Zhang et al., 2010; Zhang et al., 2012; Monsalve et al., 2009; Palaga et al., 2008; Tsao et al., 2011; Outtz et al. 2010). Moreover, we and others have shown that Notch-RBP-J is required for induction of a restricted subset of inflammatory M1 genes that includes Il12b (encodes p40 subunit shared by IL-12 and IL-23) and Nos2 (encodes iNOS). Thus, RBP-J promotes inflammatory M1 macrophage polarization in a focused manner that is important for processes such as anti-tumor responses and host defense against intracellular bacteria (Wang et al., 2010; Xu et al., 2012).

However, little is known about the role of the Notch-RBP-J pathway in M2 macrophages. As the M1 and M2 phenotypes are on extreme ends of the macrophage polarization spectrum and can oppose each other, we hypothesized that M2 responses may be augmented in RBP-J-deficient macrophages, which was supported by initial results that the M2-promoting factor JmjD3 is superinduced after TLR stimulation of RBP-J-deficient cells (Xu et al., 2012). In this study, we investigated the involvement of RBP-J in M2 macrophage polarization. Surprisingly, we found that RBP-J is critically involved in M2 polarization and function *in vivo* and *in vitro*, and regulates a restricted subset of key M2 effector molecules such as Arg1. Thus, RBP-J is the first transcription factor described to play a role in expression of transcriptional modules that are components of M1 and M2 phenotypes. This suggests that RBP-J may contribute to the complex ‘mixed’ M1-M2 phenotype that has been described *in vivo*. In addition, the common function of iNOS and Arg1 in suppressing T cell proliferation suggests an important role for RBP-J in restricting T cell proliferation in various settings of macrophage polarization.

## Results

### RBP-J promotes M2 macrophage function *in vivo*

**Figure 1:**
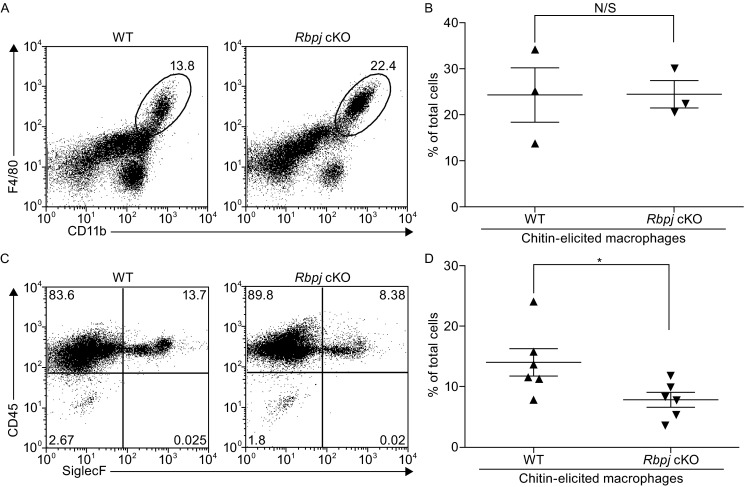
A role for *Rbpj* in eosinophil recruitment in response to chitin administration. (A) Expression of CD11b and F4/80 in peritoneal exudates cells (PECs) harvested from WT and *Rbpj* cKO mice 1 d after peritoneal injection with chitin. Circles and numbers indicate percentage of macrophages (CD11b^+^F4/80^+^) in total PECs. Results from one representative experiment are shown. (B) Cumulative data showing percentage of macrophages as in (A) from three independent experiments. (C) Expression of CD45 and SiglecF in PECs harvested from WT and *Rbpj* cKO mice 1 d after peritoneal injection with chitin. Quadrants and numbers indicate percentage of eosinophils (CD45^+^SiglecF^+^) in total PECs. (D) Cumulative data showing percentage of eosinophils as in (C) using six pairs of littermate mice with desired genotypes. Errors bars indicate s.d. *P < 0.05 (two-tailed Student’s *t*-test)

The Notch signaling pathway and RBP-J are generally thought to play a positive role in promoting classical M1 macrophage polarization (Wang et al., 2010; Xu et al., 2012). To investigate the functional roles of the Notch pathway in alternatively activated macrophage polarization, we generated mice conditionally deficient in *Rbpj* in their myeloid compartments (*Rbpj* cKO) by crossing *Rbpj*^flox/flox^ animals to animals with a *Lyz2*-Cre as described previously (Hu et al., 2008). We then subjected *Rbpj* cKO mice and wildtype (WT) littermate control animals to an *in vivo* model of chitin-induced M2 polarization. Chitin is an *N*-acetyl-β-D-glucosamine polysaccharide and a major structural component of helminthes, fungi, and anthropods. It has been previously shown that intraperitoneal administration of chitin recruits macrophages with the M2 phenotype to the peritoneal cavity, which are important for the subsequent recruitment of eosinophils (Satoh et al., 2010; Reese et al., 2007). One day after intraperitoneal administration of chitin, we found that the total number of peritoneal exudate cells (PECs) did not significantly differ between WT control and RBP-J-deficient animals (data not shown). Next, we examined populations of macrophages in PECs by flow cytometry. Consistent with previous reports, CD11b^+^F4/80^+^ macrophages were present in the PECs from WT mice (Fig. 1A). Furthermore, the percentage of CD11b^+^F4/80^+^ macrophages in PECs was comparable between WT and *Rbpj* cKO mice after chitin administration (one representative experiment shown in Fig. 1A and cumulative data from three independent experiments shown in Fig. 1B), suggesting that *Rbpj* deficiency did not significantly alter the total macrophage population in response to an M2 stimulus *in vivo*. Next, we used a well established *in vivo* model of chitin-induced functional polarization of M2 macrophages, where an important M2 function is recruitment of eosinophils to the sites of inflammation (Satoh et al., 2010; Reese et al., 2007). As measured by percentage of CD45^+^SiglecF^+^ cells as previously described (Satoh et al., 2010), chitin-induced recruitment of eosinophils was significantly reduced in *Rbpj* cKO mice compared with WT controls (one representative experiment shown in Fig. 1C and cumulative data from 6 pairs of mice shown in Fig. 1D), suggesting that *Rbpj* deficiency in the myeloid lineage impairs M2 macrophage function. Taken together, in the model of chitin-induced M2 polarization, recruitment of macrophages to peritoneal cavity does not require RBP-J, but RBP-J is indispensable for optimal functional polarization of M2 macrophages *in vivo*.

### RBP-J promotes M2 polarization *in vivo*

**Figure 2:**
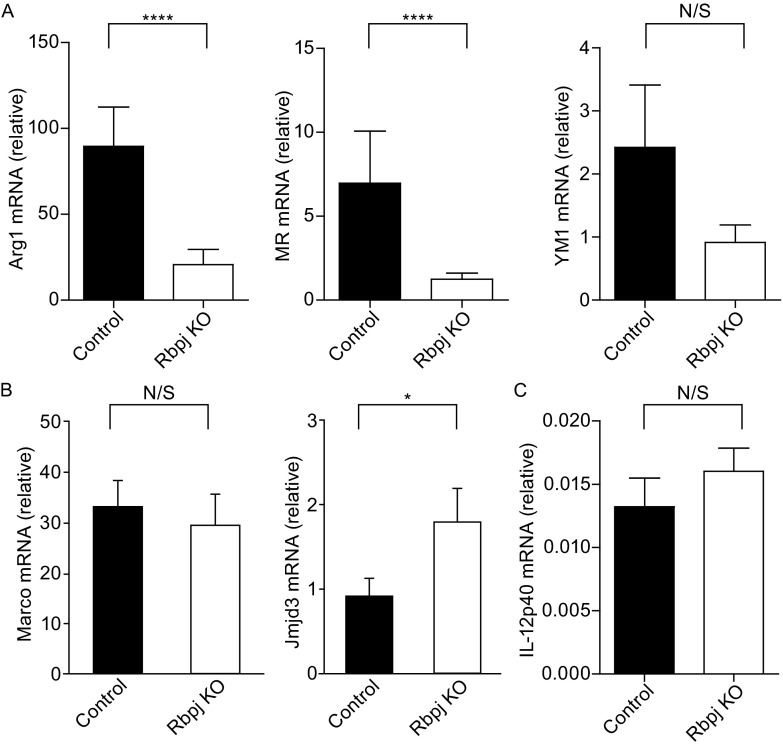
Crucial role for *Rbpj* in regulating M2 macrophage polarization in response to chitin administration *in vivo*. Total mRNA was prepared from PECs isolated from WT and *Rbpj* cKO mice 24 h after administration of chitin, and mRNA expression of Arg1, MR, Ym1, Marco, Jmjd3, and IL-12p40 (relative to GAPDH mRNA) was measured using quantitative real-time PCR. ****P < 0.0001 (two-tailed Student’s *t*-test). *P < 0.05 (two-tailed Student’s *t*-test). Results are representative of four independent experiments, each with two-four pairs of littermate mice (analyzed individually)

Next, we wished to investigate the mechanisms underlying promotion of M2 function by RBP-J and examined phenotypes of chitin-elicited macrophages. A previous report has demonstrated that after chitin injection, classical M2 markers including arginase 1 (Arg1), mannose receptor (MR), and Ym1 are mainly expressed in macrophages but not in other PECs such as B cells or eosinophils (Satoh et al., 2010). Therefore, we assessed expression of M2 macrophage-associated genes in chitin-elicited PECs and found that expression of genes encoding Arg1 and MR was significantly lower in PECs isolated from *Rbpj* cKO mice compared with WT controls (Fig. 2A). In addition, expression of mRNA encoding another M2 marker, Ym1, also showed a trend towards decreased expression in *Rbpj* cKO mice (Fig. 2A), suggesting that *Rbpj* deficiency in the myeloid cells leads to impaired M2 polarization *in vivo* as manifested by reduced expression of a subset of M2-associated genes. However, RBP-J deficiency did not result in a global defect in M2 gene program as expression of *Marco* and *Jmjd3*, two prototypical M2-associated genes was not reduced in *Rbpj* cKO mice (Fig. 2B), indicating a restricted effect on M2 phenotype that is similar to the restricted effects of RBP-J deficiency on the M1 phenotype (Xu et al., 2012). In addition, the expression of an M1 macrophage-associated gene encoding IL-12p40 subunit was unchanged after chitin administration (Fig. 2C). Of note, we and others have shown that IL-12p40 upregulation following LPS treatment is attenuated in *Rbpj*-deficient bone-marrow derived macrophages (BMDMs) (Wang et al., 2010; Xu et al., 2012). Our results shown here are not in contradiction with previously published results regarding regulation of IL-12p40 expression because chitin administration did not upregulate IL-12p40 mRNA expression in PECs and IL-12p40 levels remain near baseline and low compared to the expression of the M2 genes encoding Arg1 and MR (data not shown). These results show that RBP-J is required for development of macrophages that express Arg1 *in vivo* in the chitin-induced model of M2 polarization.

### RBP-J is required for M2-mediated suppression of T cell proliferation

**Figure 3:**
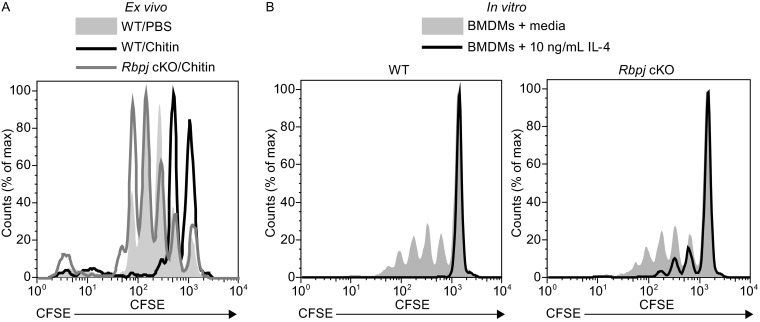
*Rbpj*-deficient alternatively activated macrophages are defective in suppressing T cell proliferation. (A) PECs from control, PBS-injected WT mice (filled gray histogram), and day-1 chitin-elicited PECs from WT mice (black line) and *Rbpj* cKO (grey line) were co-cultured with CFSE-labeled LN cells from OT-II transgenic mice and cognate OVA peptide. Proliferation of CD4^+^ cells was examined at 96 h of co-culture. Results are representative of three independent experiments. (B) CFSE-labeled OT-II transgenic LN cells were co-cultured with control (filled gray histogram) or IL-4-treated (10 ng/mL, black line) BMDMs from WT or *Rbpj* cKO mice and cognate OVA peptide. Proliferation of CD4^+^ cells was examined at 96 h of co-culture. Results are representative of two independent experiments

One important function of M2 macrophages is the suppression of T cell proliferation and expression of Arg1 is thought to be critical for M2-mediated suppression of T cells (Pesce et al., 2009). As RBP-J promotes expression of M2-associated genes including *Arg1*, we hypothesized that RBP-J may be involved in inhibition of T cell responses by M2 macrophages and assessed the role of RBP-J in mediating this M2 function both *ex vivo* and *in vitro*. For *ex vivo* experiments, we co-cultured PECs isolated from *Rbpj* cKO mice and littermate control mice following intraperitoneal chitin administration with CFSE-labeled OT-II transgenic lymph node (LN) cells in the presence of cognate OVA peptide. Consistent with previous reports (Pesce et al., 2009), we found that compared to PECs isolated from PBS-injected WT mice, chitin-elicited PECs severely inhibited CD4^+^ T cell proliferation in response to OVA (Fig. 3A, filled gray histogram versus black line), demonstrating a potent suppressive function of chitin-polarized M2 macrophages. However, when chitin-elicited PECs from *Rbpj* cKO mice were used in the co-culture, CD4^+^ T cell proliferation was comparable to that mediated by control, PBS-treated, PECs (Fig. 3A, gray line), implicating a loss of suppressive function in *Rbpj*-deficient cells and thus suggesting that RBP-J plays a crucial role in the inhibition of T cell proliferation by chitin-elicited PECs. To confirm this result, we turned to an *invitro* system of T cell suppression and used BMDMs isolated from *Rbpj* cKO and WT littermate control mice cultured in the presence of M-CSF either with or without IL-4 for 24 h to induce alternative activation before the addition of CFSE-labeled LN cells. Similar to our results with *ex vivo* PECs, we found that WT BMDMs treated with IL-4 strongly inhibited CD4^+^ T cell proliferation as compared to BMDMs cultured without IL-4 (Fig. 3B, left panel). This inhibition was attenuated when *Rbpj*-deficient BMDMs were used in the co-cultures (Fig. 3B, right panel). Taken together, these results show that RBP-J promotes M2 suppressive functions including inhibition of T cell proliferation, likely through regulation of expression of M2 effector molecules such as Arg1.

### RBP-J promotes expression of M2-associated molecules

**Figure 4:**
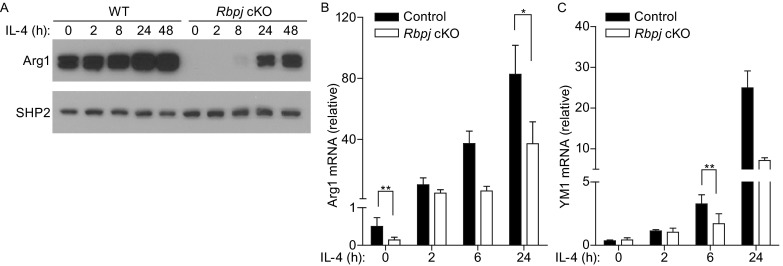
A crucial role for *Rbpj* in regulating expression of a subset of M2 macrophage genes. BMDMs from WT and *Rbpj* cKO mice were stimulated with IL-4 (10 ng/mL) for the indicated times. (A) Immunoblot analysis of whole cell lysates using antibodies recognizing Arg1. Levels of SHP2 served as loading controls. Results are representative of three independent experiments. (B and C) mRNA expression (relative to GAPDH mRNA) was measured using quantitative real-time PCR. Cumulative results from three independent experiments are shown (errors bars indicate s.d.). *P* < 0.05, **P < 0.01 (two-tailed Student’s *t*-test)

As we observed a requirement for RBP-J in maintaining high levels of M2-asscoiated genes *in vivo* in chitin-elicited PECs (Fig. 2A), we wished to further characterize RBP-J-mediated regulation of M2 genes. We utilized the *in vitro* system of M-CSF-differentiated BMDMs from *Rbpj* cKO and littermate control mice and treated the cells with IL-4 after 5 days of M-CSF-induced differentiation to induce M2 macrophage polarization. As expected, Arg1 protein and mRNA expression was induced by IL-4 in a time-dependent manner in control WT macrophages (Fig. 4A and 4B). Interestingly, *Rbpj*-deficient BMDMs expressed substantially less Arg1 protein and mRNA than BMDMs isolated from littermate controls prior to IL-4 treatment (Fig. 4A and 4B, 0 h time points). Nevertheless, *Rbpj*-deficient BMDMs were able to upregulate Arg1 mRNA and protein expression in response to IL-4 stimulation (Fig. 4A and 4B, lanes 6–10). However, due to the drastically reduced baseline expression, Arg1 expression was still markedly lower post IL-4 stimulation in *Rbpj*-deficient cells compared with Arg1 levels in control macrophages (Fig. 4A and 4B). These results implicate that RBP-J plays an essential role in maintaining baseline expression of Arg1 in macrophages but is dispensable for its IL-4-induced upregulation. In contrast, baseline mRNA expression of Ym1 was similar between WT and *Rbpj*-deficient BMDMs; however, IL-4-induced upregulation was attenuated in the absence of *Rbpj* (Fig. 4C). Taken together, our results indicate that RBP-J regulates expression of M2-associated molecules by either maintaining their basal levels in macrophages or promoting gene induction in response to M2-polarizing factors such as IL-4.

**Figure 5:**
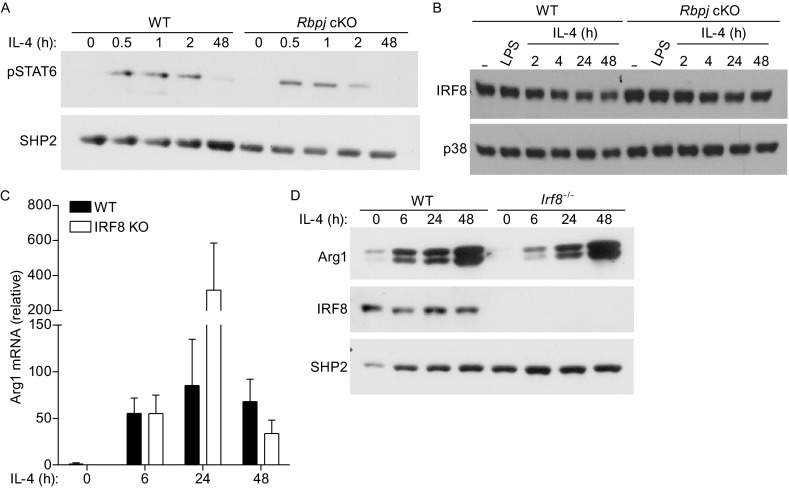
RBP-J regulates Arg1 expression in macrophages independently of STAT6 and IRF8. (A) BMDMs from WT and *Rbpj* cKO mice were stimulated with 100 ng/mL of IL-4 for the indicated times. Whole cell lysates were analyzed with immunoblotting using antibodies recognizing STAT6 phosphorylated on Tyr641 (pSTAT6). Levels of SHP2 served as loading controls. Results are representative of three independent experiments. (B) BMDMs from WT and *Rbpj* cKO mice were stimulated with 10 ng/mL of IL-4 and 1 ng/mL of LPS for the indicated times. Whole cell lysates were analyzed with immunoblotting using antibody recognizing IRF8. Levels of p38 served as loading controls. Results are representative of three independent experiments. (C) mRNA expression of Arg1 (relative to GAPDH mRNA) was measured in BMDMs from WT and *Irf8* ^−/−^ mice. Cumulative results from two independent experiments (errors bars indicate s.d.) are shown. (D) BMDMs from WT and *Irf8* ^−/−^ mice were stimulated with 10 ng/mL of IL-4 for the indicated times. Whole cell lysates were analyzed with immunoblotting using antibodies against Arg1 and IRF8. Levels of SHP2 served as loading controls. Results are representative of three independent experiments

As Arg1 is a key M2 effector molecule whose basal expression is largely dependent on RBP-J in macrophages, we wished to further elucidate the mechanisms by which RBP-J regulates Arg1 expression. It has been previously established that the *Arg1* gene expression is controlled by several transcription factors such as STAT6 and CAAT/enhancer binding protein-β (C/EBPβ) (Chawla et al., 2011; El Kasmi et al., 2008). Therefore, we examined activation and expression of these transcription factors in *Rbpj*-deficient and control macrophages. We found that activation of STAT6 as assessed by its tyrosine phosphorylation was similar in WT and *Rbpj*-deficient macrophages after IL-4 treatment (Fig. 5A), showing that IL-4-induced STAT6 signaling remains intact in the absence of RBP-J. This result is consistent with the observation that IL-4-inducible Arg1 expression is not affected by *Rbpj*-deficiency (Fig. 4A and 4B), suggesting that RBP-J is dispensable for IL-4 signaling and STAT6 activation in macrophages. In addition, C/EBPβ protein expression was unchanged in *Rbpj*-deficient macrophages (data not shown), implying that neither STAT6 nor C/EBPβ is the target of RBP-J-mediated regulation. Recently, the transcription factor, interferon regulatory factor (IRF) 8, has also been implicated in regulating Arg1 expression under various experimental conditions (Pourcet et al., 2011). We found that both at baseline and following IL-4 stimulation, IRF8 protein expression did not significantly differ between WT and *Rbpj*-deficient macrophages (Fig. 5B), suggesting that IRF8 expression is not regulated by RBP-J under these experimental conditions. Furthermore, BMDMs from *Irf8*-null mice showed normal expression and induction of Arg1 mRNA (Fig. 5C) and protein (Fig. 5D) following IL-4 stimulation, confirming that IRF8 is dispensable for IL-4-induced expression of Arg1 in macrophages. Taken together, our results indicate that RBP-J determines basal macrophage Arg1 expression but does regulate transcription factors such as STAT6, C/EBPβ, and IRF8 that induce Arg1 expression in response to environmental cues.

## Discussion

In this study we demonstrated that functional polarization of M2 macrophages *in vitro* and *in vivo* is critically regulated by a transcription factor RBP-J that integrates signals from multiple input sources including the Notch signaling pathway. RBP-J expression in myeloid cells promoted M2-mediated immune effector functions including recruitment of eosinophils and suppression of T cell proliferation by regulating expression of a subset of M2-associated genes. Our results identified RBP-J as a key regulator of M2 polarization and suggest that targeting pathways that modulate RBP-J activities has the potential of altering macrophage polarization processes and subsequent immune effector responses.

One notable feature of regulation of the M2 gene program by RBP-J is its selectivity. In contrast to many previously described M2-driving transcription factors such as IRF4 and Stat6 that promote expression of a wide-range of M2-associated genes, RBP-J only selectively enhances expression of a subset of M2 genes including those encoding Arg1 and MR while imposing minimal or even inhibitory effects on other well-characterized M2 signature genes. The highly selective nature of RBP-J on the M2 gene program is reminiscent of its role in M1 polarization, in which only a subset of M1 signature genes are regulated by RBP-J. Thus, it emerges that instead of a so-called “master transcription regulator” by a conventional definition, RBP-J serves to focus the macrophage responses on certain modules appropriate for the current microenvironment and cellular context. For example, within the broad range of various M1 effector functions, RBP-J predominantly focuses on the “bacterial host defense” module by targeting IL-12 and iNOS with minimal impact on “acute inflammation” module by sparing IL-1β. Similarly, under M2 polarizing conditions, RBP-J mainly controls eosinophil recruitment and T cell responses by targeting Arg1 while leaving Stat6-dependent processes mostly untouched. Given the highly specific and focused nature of RBP-J-mediated regulation and the fact that RBP-J integrates signals from diverse environmental cues, we propose to conceptualize RBP-J as a modular regulator of M1-M2 macrophage polarization processes whose activities can be fine-tuned in a context-dependent manner.

Given the fact that RBP-J promotes certain components of canonical M1 as well as M2 responses, it is anticipated that RBP-J would possess complex and context-dependent biological functions *in vivo* that could not be attributed as simply promoting or suppressing inflammatory and immune responses. Indeed, this is often the case. While we and others have shown that RBP-J is essential for host defense against certain intracellular bacterial species, expression of RBP-J in tumor-associated macrophages (TAMs) is suppressive for T-cell mediated anti-tumor immune responses (Franklin et al., 2014). This notion is in line with our observations that RBP-J in macrophages is required for inhibition of T cell proliferation under both *ex viv*o and *in vitro* M2-polarizing conditions (Fig. 3A and 3B) and suggests that antagonizing RBP-J activities in TAMs may represent a novel strategy of anti-tumor immune-therapy via boosting T cell responses.

In addition to biological consequences of RBP-J-mediated regulation, we also sought to investigate molecular mechanisms by which RBP-J regulated M2 gene expression especially expression of Arg1 given its role as a critical M2 effector molecule and its drastic regulation by RBP-J at the protein level (Fig. 4A). RBP-J appeared to maintain constitutive Arg1 expression in macrophages without significantly altering the capacity of macrophages to upregulating Arg1 expression in response to stimuli such as IL-4. These observations raised several interesting questions regarding mechanisms of Arg1 regulation. Firstly, is *Arg1* a direct Notch target gene or is its expression regulated by RBP-J via indirect means? These two possibilities can be distinguished by application of the Notch signaling inhibitors such as γ-secretase inhibitors or usage of Notch component-deficient animals in the future experiments. Secondly, the result that RBP-J did not regulate IL-4-activated Stat6 signaling suggests that regulation of Arg1 is Stat6-independent. Indeed, consistent with this notion, IL-4-Stat6 signaling is also dispensable for M2 effector functions in the chitin-induced model (Reese et al., 2007). Previous reports have shown that leukotriene B4 and its high-affinity receptor BLT1 are essential for chitin-induced M2 polarization (Reese et al., 2007) and it will be of great interest to investigate the involvement of leukotriene in RBP-J-mediated regulation of M2 phenotypes in the future.

## Materials and methods

### Mice

*Rbpj*^flox/flox^ mice were kindly provided by Tasuku Honjo. Mice with a myeloid-specific deletion of *Rbpj* were generated by crossing *Rbpj*^flox/flox^ animals to animals with a *Lyz2*-Cre on the C57/BL6 background as described previously (Hu et al., 2008). Mice with *Rbpj*^flox/flox^*Lyz2*-Cre genotypes were used for experiments. Gender-matched littermates with *Rbpj*^+/+^*Lyz2*-Cre or *Rbpj*^flox/+^*Lyz2*-Cre genotypes were used as controls. OT-II transgenic mice were obtained from the Jackson Laboratory. The experiments using mice were approved by the Hospital for Special Surgery and Tsinghua University Institutional Animal Care and Use Committees.

### Chitin administration

Chitin was obtained from Sigma, washed three times in PBS, and sonicated on ice. The suspended solution was filtered through a 100 μmol/L strainer and diluted in 50 mL PBS. Approximately 800 ng of chitin was intraperitoneally injected to each mouse and peritoneal exudate cells (PECs) were collected 1 d after chitin injection for flow cytometry and gene expression analysis.

### Flow cytometry

PECs were harvested and stained with antibodies specific to F4/80, CD11b, CD45, and/or SiglecF (BD). Cells were then washed three times with flow cytometry buffer. Data was acquired on a FACScan flow cytometer (BD) and analyzed using Cell Quest software (BD).

### Cells and reagents

Murine BMDMs were obtained as described and maintained in DMEM supplemented with 10% FBS and 10% L929 cell supernatant as conditioned medium providing macrophage colony stimulating factor (M-CSF). After 5 days of culture, floating cells were discarded and attached macrophages were re-plated in 12-well plates overnight prior to stimulation. Recombinant mouse IL-4 was from Peprotech and used at 10 ng/mL unless otherwise noted.

### mRNA isolation and real time PCR

RNA was extracted from whole cell lysates with an RNeasy Mini kit (Qiagen) and 0.5 μg of total RNA was reverse transcribed with a First Strand cDNA synthesis kit (Fermentas). Quantitative real time PCR (qRT-PCR) was performed in triplicate wells with an iCycler IQ thermal cycler and detection system (Biorad) using gene-specific primers. Threshold cycle numbers were normalized to triplicate samples amplified with primers specific for of glyceraldehyde-3-phosphate dehydrogenase (GAPDH).

### Proliferation assay

CFSE-labeled OT-II transgenic LN cells were co-cultured with PECs or BMDMs from WT or *Rbpj*-deficient mice and cognate OVA peptide. Proliferation of CD4^+^ cells was examined at 96 h of co-culture by assessing CFSE dilution using flow cytometry.

### Immunoblotting analysis

Whole cell lysates were prepared by direct lysis in SDS loading buffer. For immunoblot analysis, lysates were separated by 7.5% SDS-PAGE and transferred to a PVDF membrane for probing with antibody. Polyclonal antibodies against arginase 1, IRF8, p38, and Shp2 were from Santa Cruz Biotechnology. pY-Stat6 antibody was from Cell Signaling Technology.

### Statistical analysis

All statistical analyses were performed by using the Student’s *t* test.
